# Identification of Novel Tissue-Specific Genes by Analysis of Microarray Databases: A Human and Mouse Model

**DOI:** 10.1371/journal.pone.0064483

**Published:** 2013-05-31

**Authors:** Yan Song, Jinsoo Ahn, Yeunsu Suh, Michael E. Davis, Kichoon Lee

**Affiliations:** 1 Department of Animal Sciences, The Ohio State University, Columbus, Ohio, United States of America; 2 The Ohio State University Interdisciplinary PhD Program in Nutrition (OSUN), The Ohio State University, Columbus, Ohio, United States of America; University of Jaén, Spain

## Abstract

Understanding the tissue-specific pattern of gene expression is critical in elucidating the molecular mechanisms of tissue development, gene function, and transcriptional regulations of biological processes. Although tissue-specific gene expression information is available in several databases, follow-up strategies to integrate and use these data are limited. The objective of the current study was to identify and evaluate novel tissue-specific genes in human and mouse tissues by performing comparative microarray database analysis and semi-quantitative PCR analysis. We developed a powerful approach to predict tissue-specific genes by analyzing existing microarray data from the NCBI′s Gene Expression Omnibus (GEO) public repository. We investigated and confirmed tissue-specific gene expression in the human and mouse kidney, liver, lung, heart, muscle, and adipose tissue. Applying our novel comparative microarray approach, we confirmed 10 kidney, 11 liver, 11 lung, 11 heart, 8 muscle, and 8 adipose specific genes. The accuracy of this approach was further verified by employing semi-quantitative PCR reaction and by searching for gene function information in existing publications. Three novel tissue-specific genes were discovered by this approach including AMDHD1 (amidohydrolase domain containing 1) in the liver, PRUNE2 (prune homolog 2) in the heart, and ACVR1C (activin A receptor, type IC) in adipose tissue. We further confirmed the tissue-specific expression of these 3 novel genes by real-time PCR. Among them, ACVR1C is adipose tissue-specific and adipocyte-specific in adipose tissue, and can be used as an adipocyte developmental marker. From GEO profiles, we predicted the processes in which AMDHD1 and PRUNE2 may participate. Our approach provides a novel way to identify new sets of tissue-specific genes and to predict functions in which they may be involved.

## Introduction

Tissue-specific gene expression plays a fundamental role in multi-cellular biology. In general, about 100 to 200 signature genes are expressed in a specific tissue. A detailed understanding of the tissue-specific pattern of gene expression can help elucidate the molecular mechanisms of tissue development, gene function, and transcriptional regulation of biological processes [1]. Tissue-specific transcript analysis can indicate novel functions of known and unknown genes. The expression of tissue-specific genes can also be used as an indicator for many complex diseases. Examples include the tissue-specific expression of insulin signaling-related genes in diabetes, the stroma-tumor interaction-related genes in cancer, and the tissue-specific expression of mutant *IKBKAP* (inhibitor of kappa light polypeptide enhancer in B cells, kinase complex-associated protein) gene in Familial Dysautonomia [2].

Microarrays are established technologies that can provide large-scale gene expression data through measurements of transcript abundance in various tissues. Various tissue-specific expression information is available in many databases including GEO [3], ArrayExpress [4], TiGER [5], BODYMAP [6] and BioGPS [7]. The Gene Expression Omnibus (GEO) database contains gene expression profiles derived from curated GEO DataSets (GDS), which store originally submitted records obtained from common commercial arrays (Affymetrix, Agilent, Illumina, or Nimblegen). The GDS contains several thousand gene expression profiles with 4 to 70 microarrays per profile and 12,000 to 30,000 genes per microarray, comparing diverse tissues and cells of human and mouse origins under various experimental conditions.

The GeneAtlas data on the website (http://biogps.org) provide baseline expression data for the expression patterns of thousands of predicted genes, as well as known and poorly characterized genes, across more than 60 murine tissues, and over 100 human tissues. However, the data from microarray experiments represent only a starting point toward understanding the microarray-derived measurements of differential gene expression. Although huge amounts of useful data are available to scientists, there is a lack of a follow-up strategy to integrate and use these data to identify novel sets of genes that are important for each field of study. There have been no attempts to integrate these valuable databases to identify novel sets of tissue-specific genes that might have important functions in tissue growth and development.

The objective of the current study was to identify and evaluate novel tissue-specific genes across the human and mouse by performing an analysis of microarray databases and semi-quantitative PCR analysis. In the current study, we developed a unique approach to generate accurate predictions of tissue-specific genes by comparing expression profiles for various tissues across the human and mouse. The semi-quantitative PCR analysis confirmed the accuracy of our predictions. We identified 59 genes across 6 human and mouse adult tissues: 10 kidney-specific, 11 liver-specific, 11 lung-specific, 11 heart-specific, 8 muscle-specific, and 8 adipose-specific. Among them we discovered 3 novel tissue-specific genes: AMDHD1 (amidohydrolase domain containing 1) in the liver, PRUNE2 (prune homolog 2) in the heart, and ACVR1C (activin A receptor, type IC) in the adipose tissue. The processes in which PRUNE2 and AMDHD1 may participate were predicted according to the GEO profiles. Further studies have shown that ACVR1C is adipose tissue-specific and adipocyte-specific in adipose tissue, and can be used as an adipocyte developmental marker. Our approach provides a novel method for identifying novel tissue-specific genes and predicting functions in which they may be involved.

## Methods

### Data Sources and Processing

The microarray expression profiles from 6 tissues (kidney, liver, lung, heart, muscle, and adipose) were derived from the GEO DataSet (GDS) in the NCBI web site: GDS3142 for mouse and GDS596 for human. Each tissue was represented by GEO samples (GSMs) from 2 to 4 subjects (human kidney: GSM18955 and GSM18956; human liver: GSM18953 and GSM18954; human lung: GSM18949 and GSM18950; human heart: GSM18951 and GSM18952; human muscle: GSM19013 and GSM19014; human adipose: GSM18975 and GSM18976; mouse kidney: GSM252083, GSM252084, and GSM252085; mouse liver: GSM252074, GSM252075, and GSM252076; mouse lung: GSM252080, GSM252081, and GSM252082; mouse heart: GSM252113, GSM252114, and GSM252115; mouse muscle: GSM252070, GSM252071, GSM252072, and GSM252073; mouse adipose: GSM252093, GSM252094, and GSM252095).

**Figure 1:**
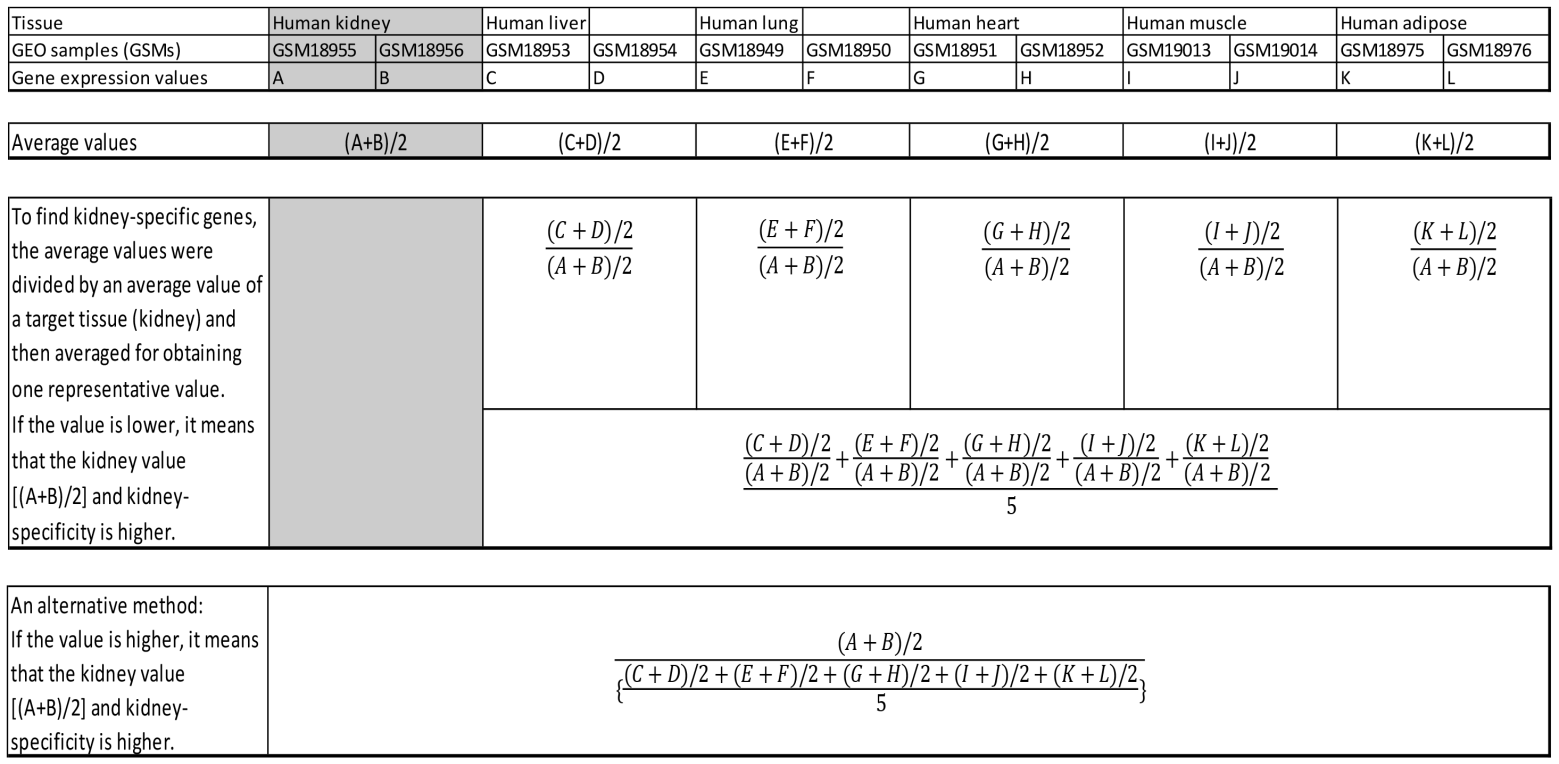
Steps for selecting kidney-specific genes. Tissue-specific genes were determined as follows: i) Gene expression values for each tissue were averaged; ii) The averaged values were divided by an averaged value for kidney; iii) The results were averaged and sorted in ascending order with a lower value representing higher tissue-specific expression. Highly ranked genes with lower values are candidates for kidney-specific genes; iv) An alternative method for finding tissue-specificity is to divide an average gene expression value for kidney by an average of averages of gene expression values in other tissues.

Tissue-specific genes were determined as follows: i) Gene expression values for each tissue in the GSM data (e.g., A and B in human kidney, C and D in human liver, and E and F in human lung) were averaged to obtain an average value [e.g., (A+B)/2, (C+D)/2, and (E+F)/2]; ii) To find tissue-specific genes (e.g., kidney-specific genes), the average values were divided by an average value of a target tissue {e.g., [(C+D)/2]/[(A+B)/2] and [(E+F)/2]/[(A+B)/2] }and then averaged to obtain one representative value <e.g., {[(C+D)/2]/[(A+B)/2]+[(E+F)/2]/[(A+B)/2]}/2>. If the value is lower, it means that the kidney value [(A+B)/2] and kidney-specificity is higher; iii) Averaged values were sorted in ascending order representing a lower value with a higher tissue-specific expression; iv) This method also shows relative gene expression ratios in other non-target tissues {e.g., [(C+D)/2]/[(A+B)/2] and [(E+F)/2]/[(A+B)/2]};v) An alternative method for finding tissue-specificity is to divide an average gene expression value for a target tissue [e.g., (A+B)/2 for kidney] by an average of averages of gene expression values in other tissues <e.g., {[(C+D)/2]+[(E+F)/2]}/2>, and sort the resulting values in descending order. Highly ranked genes shown in both human and mouse were selected for further analysis (Figure 1 shows the process of selecting kidney-specific genes; Table S1 shows the Excel spreadsheets of tissue-specific genes in selected tissues). The rank for each gene in each tissue is shown in Table S1.

### Animal Use and Ethics Statement

All animal procedures were approved by the Institutional Animal Care and Use Committee (IACUC) at The Ohio State University. All experiments were performed in accordance with the Prevention of Cruelty to Animals Act (1986). All mice were raised in a mouse housing facility at the Ohio State University and fed *ad libitum*. Mice were euthanized by CO2 inhalation followed by cervical dislocation. White adipose tissue (WAT), brown adipose tissue (BAT), liver, muscle, heart, lung, spleen, and kidney were harvested from 3-month-old mice to isolate total RNAs (n = 3). Mouse inguinal adipose tissue was collected from 1-month-old FVB mice [8].

### Differentiation of Preadipocytes

The 3T3-L1 preadipocytes were differentiated to adipocytes as previously described [8]. The 3T3-L1 preadipocytes (American Type Culture Collection, Manassas, VA, USA) were cultured in DMEM culture media (Invitrogen, Carlsbad, CA, USA) containing 10% fetal bovine serum (Invitrogen) and the mixture solution of penicillin and streptomycin (Pen Strep; Invitrogen). The preadipocytes were maintained and grown to confluence at 37°C in 5% CO_2_. After 2 d post-confluence (Day 0), differentiation of 3T3-L1 preadipocytes to adipocytes was induced by treating the preadipocytes for 2 d with a differentiation media, which contains 1 µM dexamethasone, 0.25 mM isobutylmethylxanthine, and 1 µg/ml insulin (Sigma-Aldrich Co., St. Louis, MO, USA) additionally in the culture media. Two days after induction (Day 2), the differentiation media was changed to the insulin media, which was composed of 1 µg/ml insulin in the culture media, for the next 2 d. Two days later (Day 4), the insulin media was changed to the DMEM culture media for another 4 d and the media was changed every 2 d. Total RNA was isolated from the 3T3L1 adipocytes at d 0, 2, 4, 6, and 8 post-differentiation.

### cDNA Synthesis and PCR Analysis

Mouse total RNA was isolated from kidney, liver, lung, heart, muscle, and adipose tissue of adult mice using Trizol reagent (Invitrogen; [8]). Adult human RNAs from kidney, liver, lung, heart and muscle were purchased from Agilent Technologies (Santa Clara, CA, USA) and adult human RNA from adipose tissue was bought from Clontech Laboratories (Mountain View, CA, USA). Stromal vascular (SV) and fat cell (FC) fractionation were isolated according to procedures described previously [8], [9], [10]. In brief, the adipose tissue was incubated with 3.2 mg/ml collagenase II (Sigma-Aldrich) in DMEM media for 1 h at 37°C in a vigorous shaker to separate each cell. The digested adipose tissue was filtered to remove large cell masses, and then centrifuged for 5 min at 500×g to isolate the floating FC fraction from the pellet of the SV fraction. Both SV and FC fractions were gathered for RNA isolation (n = 3).

RNA was reverse-transcribed to cDNA using moloney murine leukemia virus reverse transcriptase (Invitrogen). The PCR reaction consisted of 1 µL of cDNA, 0.5 µL of 10 mM deoxynucleoside triphosphate mix (dNTP), 2.5 µL of 10×ThermopolII (Mg-free) reaction buffer, 0.5 µL of 100 mM MgSO_4_, 0.5 nM of each of the forward and reverse primers, 0.125 µL of Taq DNA polymerase (Thermo Fisher Scientific, Waltham, MA, USA), and nuclease-free water up to 25 µL. The cycling parameters were 95°C for 5 min, followed by 25 to 30 cycles of 94°C for 30 s, 58°C for 30 s, and 72°C for 40 s with a final elongation at 72°C for 10 min. A 1% agarose gel was used to check PCR amplification.

For cDNA reverse transcription, 1 µg of total RNA, oligo dT, and M-MLV reverse transcriptase (Invitrogen) were used. The conditions for reverse transcription were 65°C for 5 min, 37°C for 50 min, and 70°C for 15 min. Quantitative real-time PCR was performed as described previously [8], [11] using AmpliTaq Gold polymerase (Applied Biosystems, Foster City, CA, USA) and SYBR green I as a detection dye. The sequences of primers for real-time PCR of cyclophilin (cyc), delta-like 1 (DLK1), fatty acid binding protein 4 (FABP4) and stearoyl-CoA desaturase-1 (SCD1) were as described previously [8], [11]. All other primers used are listed in Table 1. The mRNA expression of each gene was normalized to mRNA expression of cyclophilin, which was used as an internal control.

**Table 1:** Primer sequences for PCR amplification.

Gene name	Primer sequence (5′–3′)	Gene name	Primer sequence (5′–3′)
SCGB1A1	h-f: CCACCAGACTCAGAGACGGAAC	SLC22A2	h-m-f: GTCAGCAAAGCAGGCTGGTTA
	h-r: TGGGCGTGGACTCAAAGCA		h-m-r: GCATGATGAGGCCCTGGTA
	m-f: ACAACATCACCCCACATCTACAGAC	PDZK1	h-m-f: CTGGTCAGAAAGAGTGGGAATTCA
	m-r: CAAAGAGGAAGGAGGGGTTGG		h-m-r: TTTCAACCACTTCCTCATGGCT
SFTPB	h-m-f: ACCCTCTGCTGGACAAGCT	SLC12A3	h-m-f: ATCATTTCCAACTTCTTCCTCTGC
	h-m-r: AGCCAGGCACTGGCAGAT		h-m-r: GGTAGTTCTTGATGTGGTCTTCCAC
SFTPC	h-m-f: AGCAAAGAGGTCCTGATGGAGA	SPP1	h-f: GAAAGCCATGACCACATGGA
	h-m-r: CCAGTGGAGCCGATGGA		h-r: TGGGTTTCAGCACTCTGGTCA
CLIC3	h-m-f: GCGGCTCTTCATGGTCCT		m-f: GAAAGCCATGACCACATGGA
	h-m-r: CAGGTAGCTGTCCAGCCTGG		m-r: ACTATCGATCACATCCGACTGATC
SLC34A2	h-m-f: GCAGGCATGACCTTCATCGT	SLC34A1	h-m-f: ATGCTCAACTCCCTGCTCAAGG
	h-m-r: CGAACCAGCGATACTTGGCA		h-m-r: GCAAACCAGCGGTACTTGGC
AGER	h-m-f: AACATCACAGCCCGGATTG	FXYD2	h-f: GGACGTGGACCCGTTCTACTATG
	h-m-r: GTCTCAGGGTGTCTCCTGGTC		h-r: GGAGCTTCCTTCAGCCCAAG
TNNT2	h-m-f: ATCCCCGATGGAGAGAGAGTG		m-f: CTATGAAACCGTCCGCAAAGG
	GCCACAGCTCCTTGGCCTTC		m-r: GATGAGGCTACACATGCTCCTCA
FHOD3	h-m-f: AGACCAGAGGGAAGATGATCAC	AMDHD1	h-m-f: CCTCGAGCCAGGAAGATGTTAG
	h-m-r: CAGGGCTCTGGCTTCTTCTGG		h-m-r: TCAATTAATTCATGATGGCCTCC
NPPA	h-m-f: TAGAAGATGAGGTCATGCCCC	AMBP	h-m-f: CCTATGTGGTCCACACCAACTATG
	GTCCTTGGTGCTGAAGTTTATTC		h-m-r: GACTATGGGGAGATTGCAGGC
PLN	h-m-f: AAAGTCCAATACCTCACTCGCTC	GNMT	h-m-f: CTTTGATGCTGTCATCTGCCTTG
	GAAATGCCTCAGCAAGCACGTC		h-m-r: CAGGACGCTGTGCTGGCAC
MYH6	h-m-f: TCCGCAAACAGCTGGAGGTG	HPX	h-m-f: CGCTACTACTGCTTCCAGGGTAAC
	h-m-r: CTTCTGGTTGATGAGGCTGGTG		h-m-r: GCCTCCCTTTGTCAGGAAGAC
CSRP3	h-m-f: CAAAATGTGGAGCCTGTGAAAAG	ALB	h-m-f: TGCAACACAAAGATGACAACCC
	h-m-r: CTCTTCCCACAGATGGCACAG		h-m-r: TGCCCAGGAAGACATCCTT
RYR2	h-m-f: AGGTCTCCACTTCTTCTGTGG	APOA1	h-m-f: CGGCAGAGACTATGTGTCCCAG
	h-m-r: CCAGGCTAGGTAGAGGAAGGA		h-m-r: CTTCTGGCGGTAGAGCTCCA
ACTN3	h-f: GGCTCTCTGGAGGAGCAGATG	SLC27A5	h-m-f: TCCTGCGGTACTTGTGTAAC
	h-r: CTCGGGTCAGTACCTGGTTCTC		h-m-r: TCGAACTGCACCAGCTCAAAG
	m-f: CCCAGCCGTGACCAGACACTG	FGG	h-m-f: GGCTGGGAAATGATGAGAAGAT
	m-r: TTGGGGTCCACCATGGTCATG		h-m-r: CACAGTTGCCTTCAAACTTATC
PRUNE2	h-m-f: CTACCAGATGATTGACAGACGG	CYC	h-f: CTCCTTTGAGCTGTTTGCAG
	h-m-r: GATGATGCTCTCTGGAATGTGG		h-r: CACCACATGCTTGCCATCC
ACVR1C	h-m-f: GTTTGCCTCCTGTCCATAGC		m-f: AGCACTGGAGAGAAAGGATTTGG
	h-m-r: GGTTCCCACTTTAGGATTCTG		m-r: TCTTCTTGCTGGTCTTGCCATT

### Statistical Analysis

Statistical analysis for the tissue distribution of gene expression was performed by a mixed ANOVA model (tissues showing significant expression at α = 0.05 vs. other tissues) followed by a Fisher’s protected least significant difference test. Analysis of SV and FC fraction was performed using the Student’s *t* test at *P*<0.05. Differences among the developmental time points were compared by one-way ANOVA followed by the Tukey’s post hoc test (*P*<0.05). To compare the difference between a control and an experimental group from GEO DataSets (GDS), Student’s *t* test was conducted (*P*<0.05). In addition, one-way ANOVA followed by Tukey’s post hoc test (*P*<0.05) was performed to compare multiple treatments from GDS. All statistical analyses were conducted using SAS software (version 9.2, SAS Institute Inc., Cary, NC, USA).

## Results

### Discovery of Tissue-Specific Genes

**Figure 2:**
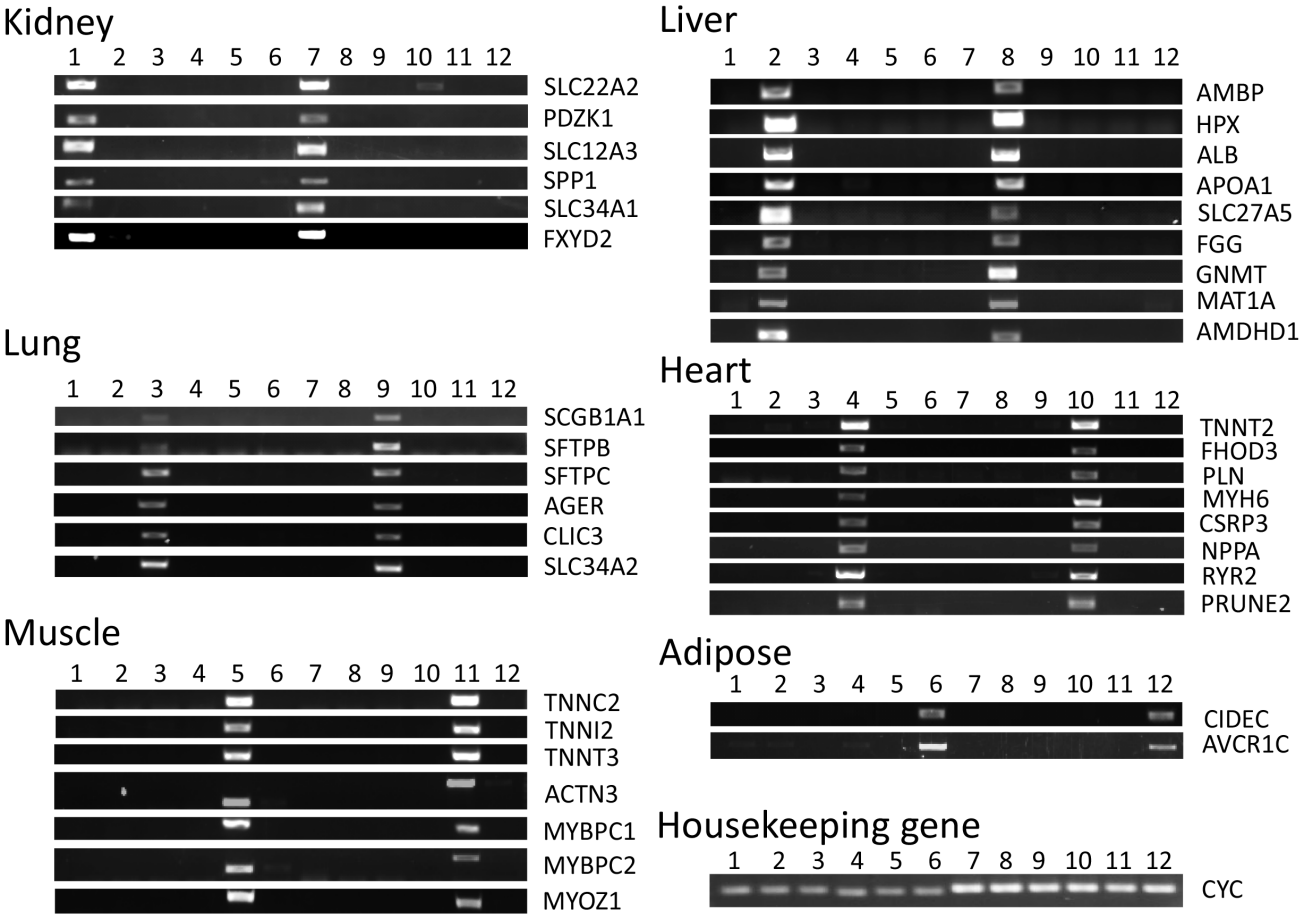
Expression of adult human and mouse gene transcripts detected by PCR reaction and agarose gel electrophoresis. Lanes 1–6 contain PCR products from human and lanes 7–12 contain PCR products from mouse. Lanes 1 and 7: kidney, lanes 2 and 8: liver, lanes 3 and 9: lung, lanes 4 and 10: heart, lanes 5 and 11: muscle, and lanes 6 and 12: adipose. Housekeeping genes, human and mouse cyclophilin (cyc), serve as a loading control.

**Figure 3:**
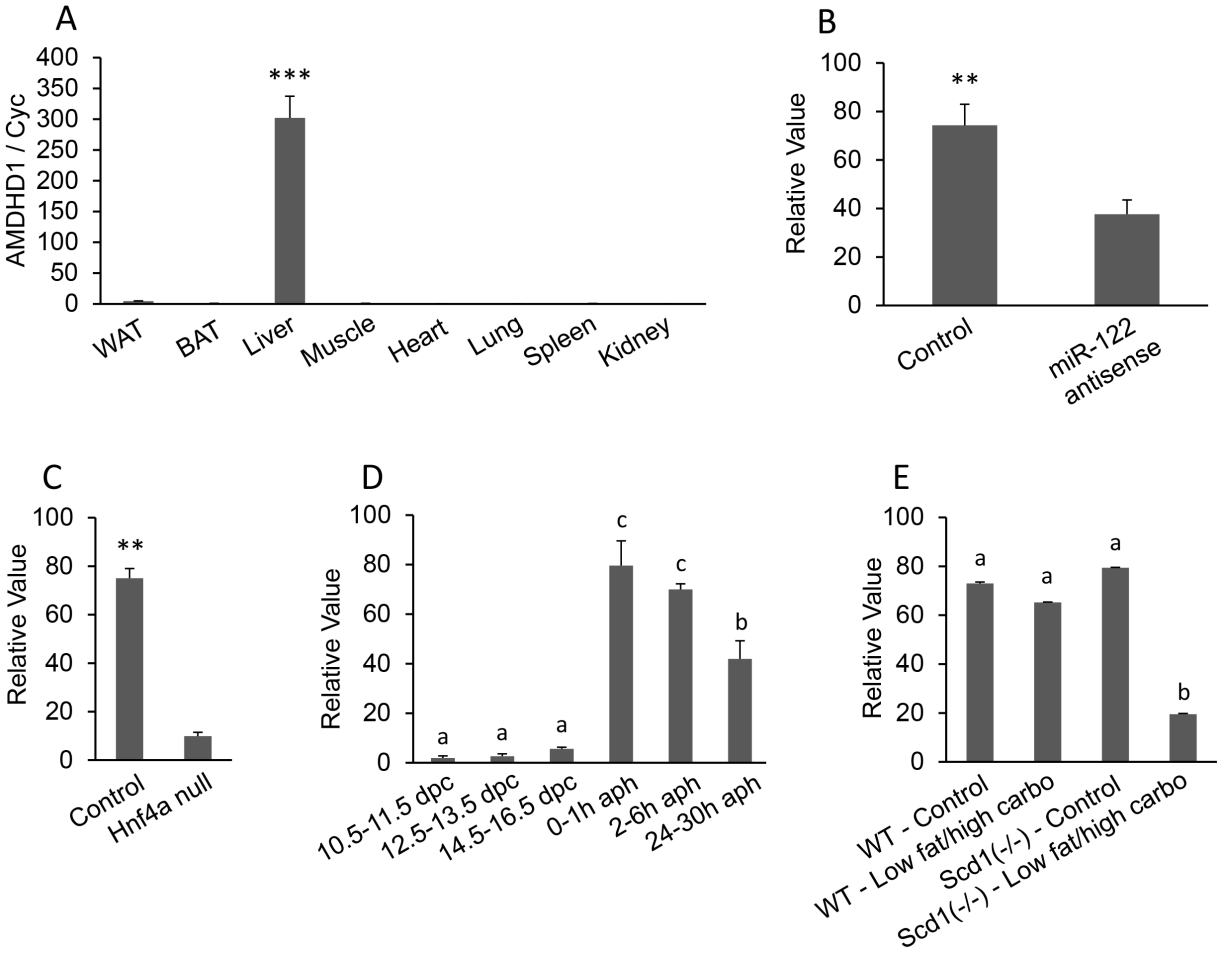
AMDHD1 (amidohydrolase domain containing 1) mRNA expression. A, Real-time PCR for AMDHD1 mRNA tissue distribution. Total RNA were isolated from the white adipose tissue (WAT), brown adipose tissue (BAT), liver, muscle, heart, lung, spleen, and kidney of adult mice. The mRNA expression was measured by quantitative real-time reverse transcription PCR (qRT-PCR) (n = 3). The bar represents mean ± SEM. Statistical significance is indicated by ***(*P*<0.001). Housekeeping gene cyclophilin (cyc) was used to normalize the mRNA expression. B–E, Analysis of microarray DataSets obtained from the NCBI website, which contains expression profiles for AMDHD1. B: GDS1729 (n = 5 per group), C: GDS1916 (n = 3 per group), D: GDS2577 (n = 3–4 per group), and E: GDS1517 (n = 5 per group). HNF4*α*: hepatocyte nuclear factor 4 alpha; dpc: days post conception; aph: after partial hepatectomy.

**Figure 4:**
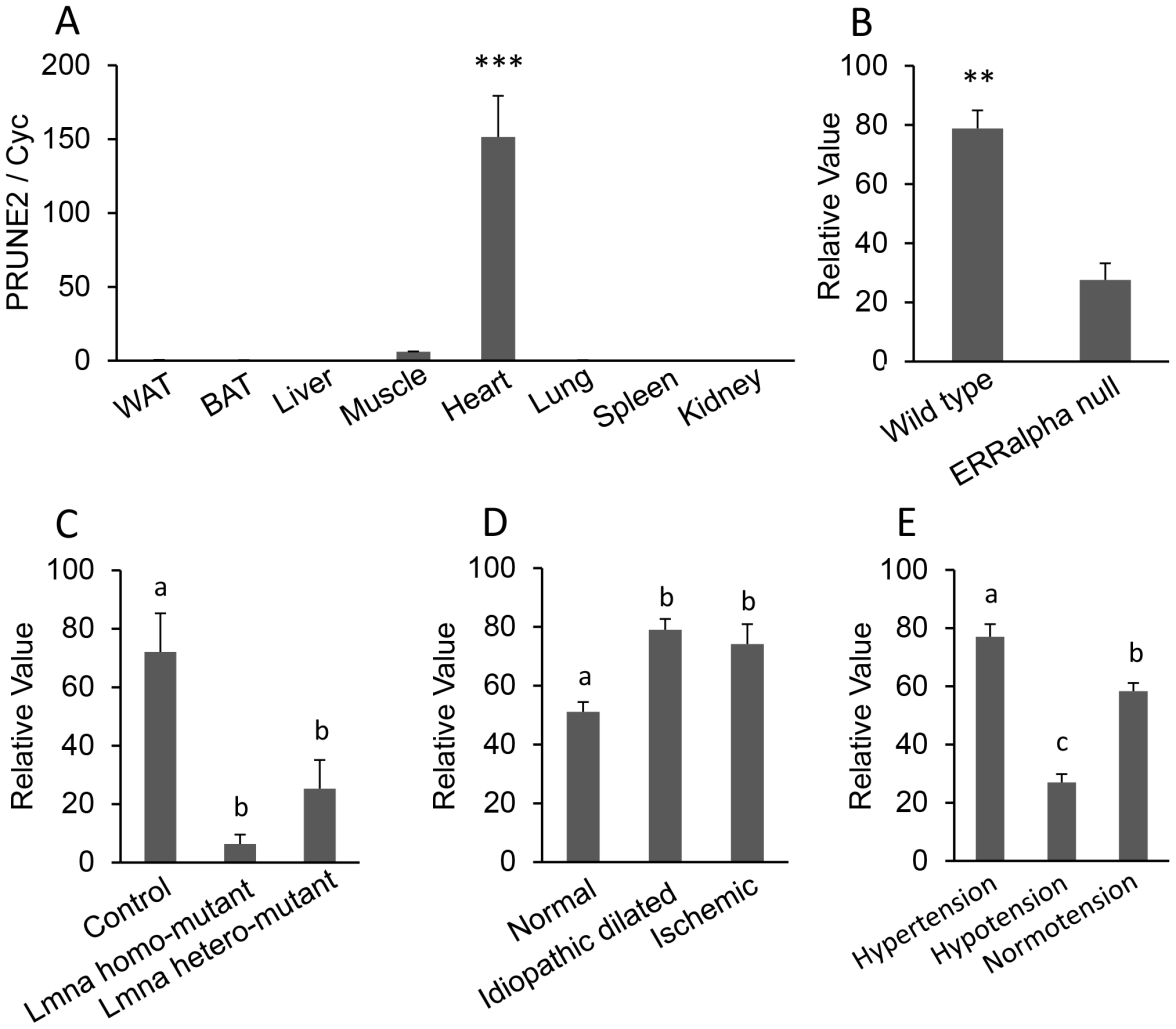
PRUNE2 (prune homolog 2) mRNA expression. A, Real-time PCR for PRUNE2 mRNA tissue distribution. Total RNA were isolated from the white adipose tissue (WAT), brown adipose tissue (BAT), liver, muscle, heart, lung, spleen, and kidney of adult mice. The mRNA expression was measured by quantitative real-time reverse transcription PCR (qRT-PCR) (n = 3). The bar represents mean ± SEM. Statistical significance is indicated by ***(*P*<0.001). Housekeeping gene cyclophilin (cyc) was used to normalize the mRNA expression. B–E, Analysis of microarray DataSets obtained from the NCBI website that contains expression profiles for PRUNE2. B: GDS2727 (n = 3 per group), C: GDS2746 (n = 6–8 per group), D: GDS651 (n = 11–15 per group), and E: GDS3673 (n = 5 per group). ERRα: estrogen-related receptor alpha; Lmna: lamin A/C.

**Figure 5:**
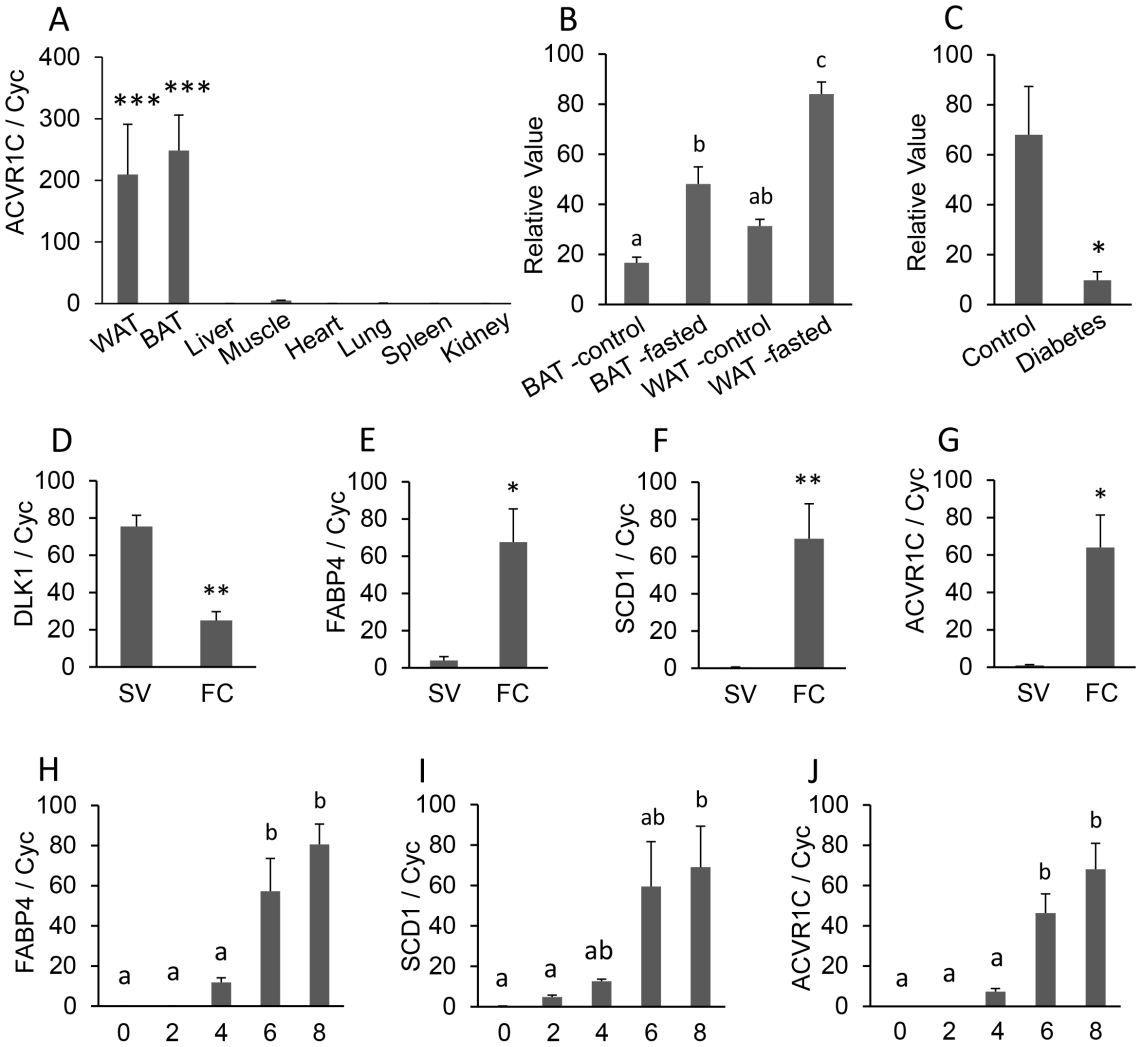
ACVR1C (activin A receptor, type IC) mRNA expression. A, Real-time PCR for ACVR1C mRNA tissue distribution. Total RNA were isolated from the white adipose tissue (WAT), brown adipose tissue (BAT), liver, muscle, heart, lung, spleen, and kidney of adult mice. The mRNA expression was measured by quantitative real-time reverse transcription PCR (qRT-PCR) (n = 3). The bar represents mean ± SEM. Statistical significance is indicated by ***(P<0.001). Housekeeping gene cyclophilin (cyc) was used to normalize the mRNA expression. B and C, Analysis of microarray DataSets obtained from the NCBI website containing expression profiles for ACVR1C. B: GDS3135 (n = 4 per group) and C: GDS3665 (n = 5 per group). D–G, Relative expression of DLK1, FABP4, SCD1, and ACVR1C in the stromal-vascular (SV) and fat cell (FC) fractions from mouse inguinal adipose tissue. Each bar indicates mean and SEM (n = 5). Statistical significance by Student’s t test is shown: *, *P*<0.05; **, *P*<0.01. The gene expression was normalized to cyclophilin (cyc) mRNA expression. H–J, Developmental regulation of ACVR1C during adipogenic differentiation of 3T3-L1 cells. The bar represents mean ± SEM (n = 3). Letters a and b show significant differences in gene expression among several time-points (day 0, 2, 4, 6, and 8) in adipocyte differentiation at *P*<0.05. The mRNA abundance was measured by quantitative real-time reverse transcription PCR (qRT-PCR) and normalized to cyclophilin (cyc) mRNA.

Twelve Excel spreadsheet files were generated for the kidney, liver, lung, heart, muscle, and adipose tissue specific expression in the human and mouse, respectively. By comparing top-rated genes across the human and mouse, 10 kidney, 11 liver, 11 lung, 11 heart, 8 muscle, and 8 adipose genes were selected. Names and ranks of selected genes, and ratios to an average of other tissues are provided in Table S1. These genes are significantly more highly expressed in certain tissues than in other tissues (*P*<0.001) with few exceptions (Table 2, 3, 4, 5, 6, 7, 8, 9, 10, 11, 12, 13). The tissue-specific expressions of those genes were confirmed either by existing publications or by semi-quantitative PCR and gel-electrophoresis (Figure 2). Three novel tissue-specific genes discovered by our method included AMDHD1 in the liver, PRUNE2 in the heart and ACVR1C in the adipose tissue. Their tissue-specific expressions were confirmed by both semi-quantitative PCR and real-time PCR (Figures 2, 3A, 4A, and 5A).

**Table 2:** Human kidney-specific gene expression values.

Gene	Kidney	Liver	Lung	Heart	Muscle	Adipose	*P* value^a^
GALNT11	1768650±25854	209850±77262	295450±8851	244800±68610	155500±30505	271400±4801	<.0001
SLC22A6	2441850±177177	41900±13602	8250±350	56900±43807	73550±12452	8250±150	<.0001
SLC22A8	4959550±860580	70850±13252	38800±10702	69900±29504	223350±60559	61700±29905	<.0001
SLC22A2	1041350±175076	136550±57659	82700±32805	41450±4651	375600±112217	31350±11652	<.0001
KL	1565900±114717	74350±53258	59600±7101	38500±27004	576700±214132	24950±19053	<.0001
PDZK1	2576450±82362	90950±68260	9000±1500	72200±35005	451500±61209	48250±1850	<.0001
SLC12A3	1107100±29805	45800±4801	19200±1600	50100±20903	130300±900	23800±10302	<.0001
SPP1	6054550±271291	112100±35005	105000±2200	59100±10902	365750±275592	140150±550	<.0001
SLC34A1	1566950±94764	268150±53958	90150±51358	142850±116768	129300±38306	60300±8701	<.0001
FXYD2	11852250±272891	155850±39056	62500±33105	265050±171576	642700±59909	99750±46257	<.0001

a *P* value represents the significance of gene expression in the human kidney compared to other tissues.

**Table 3:** Mouse kidney-specific gene expression values.

Gene	Kidney	Liver	Lung	Heart	Muscle	Adipose	*P* value^a^
GALNT11	3362±149	170±2	198±21	156±2	157±5	215±15	<.0001
SLC22A6	1986±129	105±3	110±6	157±7	118±4	103±6	<.0001
SLC22A8	1741±130	86±6	88±4	120±3	80±1	85±3	<.0001
SLC22A2	1872±59	109±5	83±3	99±4	97±3	92±6	<.0001
KL	1914±65	76±1	76±6	93±3	85±1	72±3	<.0001
PDZK1	4070±129	530±49	98±2	106±3	106±4	111±5	<.0001
SLC12A3	1987±249	136±2	120±3	143±11	120±4	115±6	<.0001
SPP1	4933±273	435±67	1037±210	104±4	145±12	104±18	<.0001
SLC34A1	7269±107	102±3	94±8	101±10	92±3	89±4	<.0001
FXYD2	9786±153	76±2	99±15	113±4	106±2	215±12	<.0001

a *P* value represents the significance of gene expression in the mouse kidney compared to other tissues.

**Table 4:** Human liver-specific gene expression values.

Gene	Kidney	Liver	Lung	Heart	Muscle	Adipose	*P* value^a^
HAMP	171900±42806	11845400±1589440	25500±20103	690200±445667	370050±119868	54150±15252	<.0001
AHSG	77800±44407	24651250±4147376	10550±150	189200±33805	318450±76662	33700±13702	<.0001
AMBP	29800±14102	44128150±5392864	9100±3601	16750±4151	65750±7251	21050±10552	<.0001
HPX	196050±100465	21364500±2278144	110450±33855	102750±33455	403000±131520	56350±3050	<.0001
ALB	511500±6101	32913750±61559	50450±9351	91800±88613	114300±38006	53000±11602	<.0001
APOA1	74300±2600	50972350±10240696	45900±11902	349250±121768	309150±136971	52800±25004	<.0001
SLC27A5	58150±12552	11243300±360854	65400±25504	38300±9101	105450±13052	35000±7401	<.0001
FGG	53400±49207	10354250±496925	74250±24254	135350±28454	333450±64160	54100±21403	<.0001
GNMT	89750±70861	4526150±933091	12350±1750	44200±30205	243850±135070	20000±8101	<.0001
MAT1A	46600±12902	8485900±318148	26350±2950	38750±14052	301400±41106	43650±8751	<.0001

a *P* value represents the significance of gene expression in the human liver compared to other tissues.

**Table 5:** Mouse liver-specific gene expression values.

Gene	Kidney	Liver	Lung	Heart	Muscle	Adipose	*P* value^a^
HAMP	82±2	6092±1249	122±4	121±16	83±3	79±4	<.0001
AHSG	93±1	8121±414	92±8	124±3	96±2	94±4	<.0001
AMBP	94±5	7279±204	79±4	101±2	89±1	82±4	<.0001
HPX	84±3	9046±350	93±5	100±1	98±3	84±1	<.0001
ALB	183±3	16668±614	106±7	139±10	120±8	142±7	<.0001
APOA1	91±4	14910±361	86±9	127±4	103±3	88±1	<.0001
SLC27A5	84±4	5640±335	87±8	115±3	94±5	80±3	<.0001
FGG	191±5	9792±373	81±1	96±4	87±4	75±4	<.0001
GNMT	485±6	9337±214	181±6	216±10	182±11	115±9	<.0001
MAT1A	76±2	4738±705	116±5	89±2	78±2	68±2	<.0001
AMDHD1	82±2	1612±126	68±6	82±3	69±3	67±4	<.0001

a *P* value represents the significance of gene expression in the mouse liver compared to other tissues.

**Table 6:** Human lung-specific gene expression values.

Gene	Kidney	Liver	Lung	Heart	Muscle	Adipose	*P* value^a^
CLDN5	247000±52608	245800±18503	3249550±98165	634400±200330	572600±375857	54650±8451	<.0001
CLDN18	158500±80912	248750±25154	2896050±202881	520000±331950	643050±209982	113450±19153	<.0001
LPCAT1	212500±131420	49550±19953	1304850±440417	27050±1650	105200±5201	90550±64060	<.001
MUC1	1190450±33755	34900±1400	3937250±418013	48050±30255	314650±164475	47250±14852	<.0001
SCGB1A1	157000±97015	131700±80312	8461200±606492	192650±121068	459000±116518	92700±16402	<.0001
SMAD6	130100±40306	48700±18203	770000±236136	193750±67260	85350±12552	23200±3901	<.001
SFTPB	130800±16002	112700±76812	18122550±2916290	128500±49708	335300±18903	143050±29955	<.0001
SFTPC	71700±3601	72400±9501	28720600±2420766	43900±8501	112150±8951	24450±3651	<.0001
AGER	54700±15702	17150±5751	1179650±770166	52950±19353	87900±21303	70850±55658	<.05
CLIC3	32700±16302	23050±5351	1270200±311047	40600±14802	108600±71411	81950±13252	<.0001
SLC34A2	252100±20203	219050±89864	2808200±206331	247100±171326	711800±399460	61950±5451	<.0001

a *P* value represents the significance of gene expression in the human lung compared to other tissues.

**Table 7:** Mouse lung-specific gene expression values.

Gene	Kidney	Liver	Lung	Heart	Muscle	Adipose	*P* value^a^
CLDN5	196±8	186±24	3131±327	259±30	227±5	721±98	<.0001
CLDN18	92±2	100±3	2162±188	113±10	88±3	82±2	<.0001
LPCAT1	179±7	147±9	2681±141	190±7	159±5	289±31	<.0001
MUC1	198±23	102±4	916±33	127±4	102±7	95±6	<.0001
SCGB1A1	92±1	73±3	16192±411	107±4	79±2	86±9	<.0001
SMAD6	165±5	134±5	1725±207	185±5	150±11	242±43	<.0001
SFTPB	146±6	148±14	8236±493	222±4	147±14	148±16	<.0001
SFTPC	100±5	105±4	17295±341	142±4	98±1	94±2	<.0001
AGER	108±4	99±3	10701±129	218±8	140±7	118±5	<.0001
CLIC3	154±4	95±3	1355±23	93±2	96±8	98±4	<.0001
SLC34A2	147±7	134±9	5913±238	265±17	137±11	104±9	<.0001

a *P* value represents the significance of gene expression in the mouse lung compared to other tissues.

**Table 8:** Human heart-specific gene expression values.

Gene	Kidney	Liver	Lung	Heart	Muscle	Adipose	*P* value^a^
FHL2	448350±147472	149000±91214	270350±7151	10829800±196430	996650±208782	545000±8801	<.0001
HSPB7	619350±7951	218500±41206	349200±7201	16034200±279242	4024050±402711	776550±73761	<.0001
MYOZ2	10800±5901	10900±3501	7650±550	1594350±58059	1238400±3501	6000±2200	<.0001
TNNT2	156200±14402	108600±35405	46200±3601	9868550±1550184	423300±216733	113600±47107	<.0001
FHOD3	285850±26454	36500±11702	34850±17253	817700±2100	171100±65210	47300±36405	<.0001
PLN	37900±16302	40200±14302	14900±7701	1052750±251388	503800±348953	15950±2550	<.005
MYH6	17600±2700	28700±4801	53050±15452	5613450±320798	1098450±264690	12700±7001	<.0001
CSRP3	12150±4151	11600±2600	5300±400	5745250±515928	2571250±283193	8150±1850	<.0001
NPPA	872650±221483	182900±79512	138900±74211	5658450±205681	2447950±762865	336900±52408	<.0001
RYR2	72050±61359	28600±5401	20250±12952	291400±22103	99800±10502	10450±3951	<.0005
PRUNE2	126700±42006	71650±20053	16850±12052	127800±46207	86450±36956	255800±8601	<.5

a *P* value represents the significance of gene expression in the human heart compared to other tissues.

**Table 9:** Mouse heart-specific gene expression values.

Gene	Kidney	Liver	Lung	Heart	Muscle	Adipose	*P* value^a^
FHL2	279±7	140±5	173±6	8829±429	161±2	146±7	<.0001
HSPB7	94±1	97±7	154±16	4441±118	1016±181	116±1	<.0001
MYOZ2	80±3	96±2	193±14	8934±199	2157±145	82±1	<.0001
TNNT2	74±4	79±4	422±27	16112±248	113±5	66±2	<.0001
FHOD3	346±22	82±2	81±4	1353±58	421±36	75±10	<.0001
PLN	102±3	75±8	185±8	14565±615	112±8	86±9	<.0001
MYH6	71±2	73±2	839±124	19317±1430	83±2	87±12	<.0001
CSRP3	86±2	411±5	247±8	8180±256	1294±176	86±0	<.0001
NPPA	77±4	88±7	261±19	4431±361	83±3	80±2	<.0001
RYR2	70±1	69±3	128±6	2889±51	74±3	68±2	<.0001
PRUNE2	72±1	77±3	74±0	215±26	85±1	83±2	<.0001

a *P* value represents the significance of gene expression in the mouse heart compared to other tissues.

**Table 10:** Human muscle-specific gene expression values.

Gene	Kidney	Liver	Lung	Heart	Muscle	Adipose	*P* value^a^
MYOT	12900±6001	19500±7601	17250±12752	92450±35755	5427150±568736	14050±4651	<.0001
TNNC2	200500±7901	85800±14002	139350±32055	81450±13252	72503600±4693109	64650±21753	<.0001
TNNI2	40300±8301	25100±5901	28150±7951	43850±450	25906100±111517	22500±9601	<.0001
TNNT3	220450±83863	228450±94964	161100±14102	565100±373356	11062700±366455	149900±32805	<.0001
ACTN3	221850±24654	198850±90564	166450±15552	256300±87213	7269400±844828	163550±40656	<.0001
MYBPC1	179500±64710	158700±64710	57750±10352	279550±101765	20410650±63360	113800±3100	<.0001
MYBPC2	16600±3701	24550±8851	6800±100	50450±5851	12523950±251088	14650±6751	<.0001
MYOZ1	311100±176027	238550±67760	180600±6901	372150±141771	15847500±217533	148900±12202	<.0001

a *P* value represents the significance of gene expression in the human muscle compared to other tissues.

**Table 11:** Mouse muscle-specific gene expression values.

Gene	Kidney	Liver	Lung	Heart	Muscle	Adipose	*P* value^a^
MYOT	66±1	65±2	63±0	836±14	7300±174	64±3	<.0001
TNNC2	82±3	94±7	80±3	135±3	15148±298	90±2	<.0001
TNNI2	89±4	94±8	105±13	109±4	15552±388	92±4	<.0001
TNNT3	79±4	101±11	90±4	83±4	14045±248	83±3	<.0001
ACTN3	92±3	107±5	101±20	119±4	11726±449	150±8	<.0001
MYBPC1	89±2	95±3	85±2	97±3	3414±295	87±4	<.0001
MYBPC2	83±2	82±4	100±2	183±13	9194±118	94±5	<.0001
MYOZ1	139±6	161±6	157±50	214±14	7523±84	145±11	<.0001

a *P* value represents the significance of gene expression in the mouse muscle compared to other tissues.

**Table 12:** Human adipose-specific gene expression values.

Gene	Kidney	Liver	Lung	Heart	Muscle	Adipose	*P* value^a^
RETN	59850±32055	86150±42056	437450±156374	174450±100065	211200±89313	73500±32705	<.5
ADIPOQ	173600±64710	141750±17753	34000±17103	393850±11152	299050±41256	10250050±1283044	<.0001
LEP	13250±3351	8900±400	8950±1250	26550±5151	78650±15952	24350±2450	<1
PPARG	42850±7951	35750±14352	80400±21503	22600±7001	84600±1400	917150±14252	<.0001
CIDEC	546300±145022	787350±256989	400000±153723	894150±271191	1235150±349403	10546600±390859	<.0001

a *P* value represents the significance of gene expression in the human adipose tissue compared to other tissues.

**Table 13:** Mouse adipose-specific gene expression values.

Gene	Kidney	Liver	Lung	Heart	Muscle	Adipose	*P* value^a^
RETN	168±13	136±2	240±8	190±18	254±44	11518±177	<.0001
ADIPOQ	177±39	67±0	355±30	123±10	830±177	15125±409	<.0001
LEP	74±2	80±1	75±2	88±4	105±10	1719±137	<.0001
PPARG	108±3	168±24	148±4	172±16	135±8	2016±159	<.0001
CIDEC	83±6	88±7	132±4	82±5	123±12	5175±484	<.0001
CCDC80	146±2	191±10	362±2	734±34	409±46	6447±167	<.0001
DGAT2	739±27	1252±158	213±8	727±29	330±16	4503±922	<.0001
ACVR1C	58±2	61±2	63±2	60±2	68±2	776±208	<.0001

a *P* value represents the significance of gene expression in the mouse adipose tissue compared to other tissues.

### Regulation of Novel Gene Expression Under Different Physiological Conditions and Disease Models

Gene expression under different physiological conditions and disease models was investigated using GEO profiles to help understand potential functions of the novel genes. The GEO profiles provided information to predict the function of AMDHD1 in the liver. GDS1729 in the GEO Profiles shows that microRNA miR-122 antisense inhibits the expression of AMDHD1 in the liver (Figure 3B, *P*<0.01). The miR-122 down-regulates target mRNAs to establish tissue-specific gene expression patterns. The decreased expression of AMDHD1 in miR-122 treated liver indicated that AMDHD1 is one of the target genes of miR-122 and is involved in liver development and formation. GDS1916 shows that the expression of AMDHD1 is decreased significantly when hepatocyte nuclear factor 4 alpha (HNF4*α*) is deleted (Figure 3C, *P*<0.01). HNF4α is a promoter for the expression of hundreds of metabolism-related genes in the liver. The co-expression of AMDHD1 and HNF4α indicates that AMDHD1 may be involved in hepatogenesis. The relatively greater expression of AMDHD1 in the regenerating liver compared to the developing liver indicates the role of AMDHD1 in renewal and repair of the liver (GDS2577; Figure 3D, *P* *<*0.05). Expression of AMDHD1 in control mice is not influenced by a low fat, high carbohydrate diet, whereas SCD1 null mice treated with a low fat, high carbohydrate diet have significantly decreased expression of AMDHD1 (GDS-1517; Figure 3E, *P*<0.05). Because SCD1 is the rate-limiting enzyme that catalyzes the synthesis of monounsaturated fatty acids [12], this result indicates that AMDHD1 may be involved in fatty acid metabolism, which is one of the main functions of the liver.

PRUNE2 was previously studied in the central nervous system and in cancer patients, but there are no reports related to the function of PRUNE2 in the heart. GEO profiles provide some information to predict the function of PRUNE2 in the heart. The expression of PRUNE2 is decreased in ERRα (estrogen-related receptor alpha) deficient mouse hearts (GDS2727; Figure 4B; *P*<0.01). ERRα regulates cellular energy metabolism, which is pivotal in high energy demand tissues such as the heart. The decreased expression of PRUNE2 in the heart of ERRα deficient mice indicates the function of PRUNE2 may be related to energy metabolism in the heart. Bonne et al. (1999) identified Lmna (lamin A/C) mutations in the autosomal dominant form of Emery–Dreifuss muscular dystrophy (AD-EDMD) [13]. The decreased expression of PRUNE2 in the Lmna H222P mutants (GDS 2746; Figure 4C; *P*<0.05) suggests a relationship between PRUNE2 and Emery–Dreifuss muscular dystrophy. Patients with idiopathic dilated heart failure and ischemic heart failure have increased expression of PRUNE2, which also indicates a role of PRUNE2 in heart function (GDS651, Figure 4D; *P*<0.05). PRUNE2 is also related to heart tension with higher expression in the heart of the hypertensive blood pressure high (BPH) inbred strains of mice and lower expression in genetically hypotensive blood pressure low (BPL) inbred strains compared to the normotensive blood pressure normal (BPN) inbred strains (GDS3673; Figure 4E; *P*<0.05). All of these data suggest that PRUEN2 is related to heart function and heart disease.

ACVR1C, also named activin receptor-like kinase 7 (ALK7), has a known ligand, Nodal, and is one of the type I transforming growth factor-β (TGF-β) receptors [14]. GDS 3135 shows that, in fasted rats, the expression of ACVR1C increased significantly in both WAT and BAT (Figure 5B), suggesting a role for ACVR1C in releasing fats in both WAT and BAT. GDS3665 shows that the expression of ACVR1C is decreased significantly (*P*<0.05; Figure 5C) in the adipose tissue from obese diabetic women compared to control women, which indicates that ACVR1C may be a contributing factor to obese diabetic symptoms.

### ACVR1C is BAT and WAT-Specific

Given that average relative expression in other tissues such as liver, muscle, heart, lung, spleen, and kidney was approximately 1, the mRNA expression of ACVR1C in BAT and WAT was about 250-fold and 210-fold higher than in other tissues, respectively (Figure 5A). Therefore, ACVR1C showed significantly higher expression in both BAT and WAT. In addition, fractionation of SV cells and fat cells was verified by predominant expression of DLK1 (a preadipocyte marker) in SV cells (P<0.01), and FABP4 and SCD1 (adipocyte markers) in fat cells (*P*<0.05 and *P*<0.01, respectively). In SV cells, mRNA expression of ACVR1C was significantly reduced. However, in fat cells, mRNA expression of ACVR1C was enhanced approximately 60-fold compared to expression in SV cells. Taken together, these results suggest that ACVR1C expression is specific to adipose tissue and adipocytes in the fat cell fraction.

### Developmental Regulation of ACVR1C in 3T3-L1 Cells

Developmental regulation of gene expression of ACVR1C has been evaluated during adipogenic differentiation of 3T3-L1 preadipocytes (Figure 5J). The development of adipocytes was demonstrated by gradual increases in the expression of adipocyte markers, FABP4 and SCD1, during differentiation. In addition, expression of both FABP4 and SCD1 increased significantly after d 6 (*P*<0.05). During 3T3-L1 preadipocyte differentiation, expression of ACVR1C showed a highly correlated pattern of expression to that of FABP4, with a significant increase at d 6 (*P*<0.05).

## Discussion

We have established a novel and powerful approach to predict tissue-specific genes. By comparing one human and one mouse GEO DataSet (GDS) from a microarray, we identified a total of 59 tissue-specific or tissue-related genes in the kidney, liver, lung, heart, muscle, and adipose tissue. After confirmation of the tissue-specific expression by semi-quantitative PCR, we searched for the functions of these genes in specific tissues using NCBI PubMed and GEO profiles to further support our approach. The following tissue-specific genes selected from a microarray were categorized as follows: i) Genes that are verified as tissue-specific in human in previous publications; ii) Genes that are verified as tissue-specific in mouse in previous publications; iii) Genes that are verified as tissue-specific in both human and mouse in previous publications; iv) Genes that are not confirmed as tissue-specific, but are said to contain tissue-related functions in previous publications; and v) Novel genes that have not been previously reported as tissue-specific or tissue-related, but are verified as tissue-specific by our PCR (Table 14, 15, 16, 17, 18, 19).

**Table 14:** Kidney-specific expression confirmed by publications and/or semi-quantitative PCR.

Kidney	Publicationin human	Publicationin mouse	PCR
GALNT11	[15]	[15]	–
SLC22A6	[16], [17]	[16]	–
SLC22A8	[18]	[19]	–
SLC22A2	[20]	[21]	
KL	[22]	[23]	–
PDZK1	–	[24]	○
SLC12A3	[25]	–	○
SPP1	–	–	○
SLC34A1	–	–	○
FXYD2	–	–	○

**Table 15:** Liver-specific expression confirmed by publications and/or semi-quantitative PCR.

Liver	Publicationin human	Publicationin mouse	PCR	
HAMP	[32]	[33]	–	
AHSG	[34]	[34]	–	
AMBP	–	[35]	○	
HPX	–	–	○	
ALB	–	–	○	
APOA1	–	–	○	
SLC27A5	–	–	○	
FGG	–	–	○	
GNMT	–	–	○	
MAT1A	–	–	○	
AMDHD1	–	–	○	Novel

**Table 16:** Lung-specific expression confirmed by publications and/or semi-quantitative PCR.

Lung	Publicationin human	Publicationin mouse	PCR
CLDN5	[47]	[48]	–
CLDN18	[49]	–	–
LPCAT1	[50]	[51]	–
MUC1	[52]	[53]	–
SCGB1A1	[54]	–	○
SMAD6	[55]	[55]	–
SFTPB	–	–	○
SFTPC	–	–	○
AGER	–	–	○
CLIC3	–	–	○
SLC34A2	–	–	○

**Table 17:** Heart-specific expression confirmed by publications and/or semi-quantitative PCR.

Heart	Publicationin human	Publicationin mouse	PCR	
FHL2	[59]	[60]	–	
HSPB7	[61]	[62]	–	
MYOZ2	[63]	[63]	–	
TNNT2	–	–	○	
FHOD3	–	–	○	
PLN	–	–	○	
MYH6	–	–	○	
CSRP3	–	–	○	
NPPA	–	–	○	
RYR2	–	–	○	
PRUNE2	–	–	○	Novel

**Table 18:** Muscle-specific expression confirmed by publications and/or semi-quantitative PCR.

Muscle	Publicationin human	Publicationin mouse	PCR
MYOT	[74]	[75]	–
TNNC2	–	–	○
TNNI2	–	–	○
TNNT3	–	–	○
ACTN3	–	–	○
MYBPC1	–	–	○
MYBPC2	–	–	○
MYOZ1	–	–	○

**Table 19:** Adipose-specific expression confirmed by publications and/or semi-quantitative PCR.

Adipose	Publicationin human	Publicationin mouse	PCR	
RETN	[80]	[81], [82]	–	
ADIPOQ	[83]	–	–	
LEP	[84]	[85]	–	
PPARγ	[86]	[87]	–	
CIDEC	[88]	–	○	
CCDC80	[89]	[89]	–	
DGAT2	[90]	[90]	–	
ACVR1C	–	–	○	Novel

### Kidney-Specific Genes

The 10 kidney-specific genes that we identified are GALNT11 ((UDP-N-acetyl-alpha-D-galactosamine:polypeptide N-acetylgalactosaminyltransferase 11), SLC22A6 [solute carrier family 22 (organic anion transporter), member 6], SLC22A8 [solute carrier family 22 (organic anion transporter), member 8], SLC22A2 [solute carrier family 22 (organic cation transporter), member 2], KL (klotho), PDZK1 (PDZ domain containing 1), SLC12A3 [solute carrier family 12 (organic cation transporter), member 3], SPP1 (secreted phosphoprotein 1), SLC34A1 [solute carrier family 34 (sodium phosphate), member 1], and FXYD2 (FXYD domain containing ion transport regulator 2). Among them, genes that have been verified by publications for the human and/or mouse include GALNT11 [15], SLC22A6 [16], [17], SLC22A8 [18], [19], SLC22A2 [20], [21], KL [22], [23], PDZK1 [24], and SLC12A3 [25]. Genes confirmed by PCR include SLC22A2, PDZK1, SLC12A3, SPP1, SLC34A1, and FXYD2.

The SLC genes belong to the solute carrier family. The SLC22A2, SLC22A6, and SLC22A8 genes are members of the organic anion transporter SLC22 gene family [26]. SLC12A3 belongs to an electroneutral cation-chloride-coupled cotransporter gene family. SLC34A1 is a member of the type II sodium-phosphate co-transporter family. All of these genes are responsible for solute (organic anion, sodium/chloride, and phosphate, respectively) transportation in the kidney, which is the key aspect for kidney function.

KL is a single transmembrane protein that is mainly produced in the kidney and brain. Severely reduced production of KL can induce chronic renal failure in the human kidney [27]. FXYD2 is the gamma subunit of the Na,K-ATPase and functions in regulating the enzyme’s activity by inducing ion channel activity. Mutations in this gene have been associated with renal hypomagnesaemia [28]. SPP1 is synthesized by the kidney and secreted into the urine by epithelial cells [29]. It functions to inhibit the nucleation and aggregation of calcium oxalate crystals [30]. PDZK1 is a scaffold protein that is located in brush borders of proximal tubular cells [24]. PDZK1 binds to and mediates the localization of cell surface proteins and plays an important role in cholesterol metabolism [31]. GALNT11 is an enzyme that is highly expressed in the kidney and catalyzes O-linked oligosaccharide biosynthesis and transfers N-acetyl-D-galactosamine residue to a serine or threonine residue on the protein receptor.

### Liver-Specific Genes

HAMP (hepcidin antimicrobial peptide), AHSG (alpha-2-HS-glycoprotein), AMBP (alpha-1-microglobulin/bikunin precursor), HPX (hemopexin), ALB(albumin), APOA1 (apolipoprotein A-I), SLC27A5 [solute carrier family 27 (fatty acid transporter), member 5], FGG (fibrinogen gamma chain), GNMT (glycine N-methyltransferase), MAT1A (methionine adenosyltransferase I, alpha), and AMDHD1 (amidohydrolase domain containing 1) exhibit significantly higher expression in liver than in other tissues. The liver-specific expressions of HAMP [32], [33], AHSG [34], and AMBP [35] in human and/or mouse were confirmed by previous publications. AMBP, HPX, ALB, APOA1, SLC27A5, FGG, GNMT, and MAT1A were confirmed as liver-specific by semi-quantitative PCR.

HAMP functions in the maintenance of iron homeostasis, and is required for intestinal iron absorption and iron storage in macrophages. Mutations in this gene may cause hemochromatosis type 2B, which is an endocrine liver disease. HPX is a plasma glycoprotein that can bind and transport heme from the plasma to the liver for iron recovery to prevent heme-mediated oxidative damage and heme-bound iron loss [36]. AHSG and ALB are both synthesized by hepatocytes and secreted to the serum. AHSG is involved in ectopic calcium deposition [37], insulin resistance [38], [39], and fat accumulation in the liver [40]. ALB composes about half of the blood serum protein. It serves as a carrier for steroids, fatty acids, thyroid hormones, and drugs and as a regulator for the colloidal osmotic pressure of blood. ALB levels are decreased in chronic liver disease and nephrotic syndrome.

AMBP is a liver-specific precursor protein of alpha-1-microglobulin and bikunin. Alpha-1-microglobulin belongs to the lipocalin transport protein superfamily and functions in inflammatory processes, whereas bikunin is a urinary trypsin inhibitor. FGG is a blood-borne glycoprotein that can be cleaved by thrombin to form fibrin and act as a co-factor in platelet aggregation. Defects in this gene lead to several disorders, including dysfibrinogenemia, hypofibrinogenemia, and thrombophilia. APOA1 is the main component of high density lipoprotein (HDL) in blood plasma. It promotes reverse transport of cholesterol from tissues to the liver for excretion by promoting cholesterol efflux from tissues [41]. SLC27A5, GNMT, and MATA1A have enzymatic activities. SLC27A5 is a fatty acid transporter, and encodes Acyl-CoA synthetase, which is involved in bile acid metabolism in the liver. GNMT catalyzes the conversion of S-adenosyl-methionine (SAM) to S-adenosylhomocysteine and sarcosine. MAT1A catalyzes the formation of S-adenosylmethionine from methionine and ATP. All of these activities occur predominantly in the liver and are important for normal liver functions.

AMDHD1 is only contained in mouse GDS and is highly ranked, indicating its liver-specific expression. Human GDS596 only contains data for about 22,000 spots, whereas the mouse GDS3142 has data for more than 45,000 spots. Searching for “AMDHD1 liver” in NCBI PubMed (http://www.ncbi.nlm.nih.gov/pubmed) returns no results. Searching GEO profiles provides indications about the function of AMDHD1 in the liver. AMDHD1 protein contains 426 amino acids and has been reported to be involved in the histidine metabolism pathway [42]. The expression of AMDHD1 in the liver is inhibited by microRNA miR-122 antisense. The miR-122 makes up 70% of all microRNA in the adult liver. It is highly expressed in the developing and adult liver [43]. It negatively regulates target mRNAs and is thought to be important for establishing tissue-specific gene expression patterns. The expression of AMDHD1 is negatively regulated by miR-122 in the liver, suggesting that AMDHD1 may be involved in liver development and formation. AMDHD1 does not have miR-122 binding sites (www.microrna.org), which indicates that miR-122 is a *trans*-acting factor for AMDHD1. HNF4α is a nuclear receptor that can activate the expression of hundreds of genes in the liver, especially metabolism-related genes in glucose, fatty acid, cholesterol, and drug metabolism [44], [45]. The HNF4α null mice are embryonic lethal [46]. The decreased expression of AMDHD1 in HNF4α deleted liver suggests that AMDHD1 is involved in hepatogenesis. GDS2577 shows that expression of AMDHD1 is significantly higher in the regenerating liver than in the developing liver, which suggests a function for AMDHD1 in renewal and repair of the liver.

SCD1 is an enzyme that is responsible for forming a double bond in stearoyl-CoA to form monounsaturated fatty acid from saturated fatty acid [12]. A low fat, high carbohydrate diet may cause SCD1 null mice to develop severe hypercholesterolemia. The significantly lower expression of AMDHD1 in the low fat, high carbohydrate treated SCD1 null mice (GDS-1517) indicates that AMDHD1 is involved in fatty acid metabolism in the liver.

### Lung-Specific Genes

Lung-specific genes identified in the current study include LPCAT1 (lysophosphatidylcholine acyltransferase 1), MUC1 (mucin 1, cell surface associated), SCGB1A1 [secretoglobin, family 1A, member 1 (uteroglobin)], SFTPB (surfactant protein B), SFTPC (surfactant protein C), AGER (advanced glycosylation end product-specific receptor), CLDN5 (claudin 5), CLDN18 (claudin 18), SLC34A2 [solute carrier family 34 (sodium phosphate), member 2], SMAD6 (SMAD family member 6), and CLIC3 (chloride intracellular channel 3). Among them, lung-specific expression in human and/or mouse was either confirmed by publications {CLDN5 [47], [48], CLDN18 [49], LPCAT1 [50], [51], MUC1 [52], [53], SCGB1A1 [54], and SMAD6 [55]} or PCR (SCGB1A1, SFTPB, SFTPC, AGER, SLC34A2, and CLIC3).

Bridges et al. (2010) reported that LPCAT1 functions in surfactant phospholipid synthesis and is essential for transitioning to air breathing in neonatal mice [56]. MUC1 serves as a protective layer in the airway against bacterial and enzyme attack. SCGB1A1 is an anti-inflammatory agent that decreases systemic inflammation and increases surfactant protein and vascular endothelial growth factor expression. It functions in reducing lung injury, improves pulmonary compliance and oxygenation. SFTPB and SFTPC are both expressed on the pulmonary surfactant to promote alveolar stability by reducing air-liquid interface tension.

The CLDNs are located on tight junction strands on the cell membrane of the lung and serve as a physical barrier for solutions and water. Mutations in CLDN5 may cause velocardiofacial syndrome [47], whereas mutations in CLDN18 are related to lung adenocarcinomas [57]. AGER is highly expressed in the embryonic brain and adult lung. AGER expression is significantly decreased in human lung carcinomas, which suggests that AGER may function in suppressing lung cancer. SLC34A2 is a phosphate transport protein. Mutations in SLC34A2 may cause pulmonary alveolar microlithiasis [58].

SMAD6 inhibits transforming growth factor-beta (TGF-β) superfamily-regulated cell growth and development. CLIC3 is a component of chloride ion channels. The functions of these two genes are understudied in the lung. By searching the GEO profile, we predict that SMAD6 and CLIC3 could be related to idiopathic pulmonary fibrosis and pulmonary adenocarcinomas. Compared to the normal lung tissue, the expressions of SMAD6 and CLIC3 are lower in tissues with idiopathic pulmonary fibrosis (GDS1252) and pulmonary adenocarcinoma (GDS1650 and GDS 3257).

### Heart-Specific Genes

In the heart, FHL2 (four and a half LIM domains 2), HSPB7 [heat shock 27 kDa protein family, member 7 (cardiovascular)], MYOZ2 (myozenin 2), FHOD3 (formin homology 2 domain containing 3), PLN (phospholamban), MYH6 (myosin, heavy chain 6, cardiac muscle, alpha), CSRP3 [cysteine and glycine-rich protein 3 (cardiac LIM protein)], NPPA (natriuretic peptide A), RYR2 (ryanodine receptor 2, cardiac), TNNT2 (troponin T type 2, cardiac), and PRUNE2 have significantly higher expressions compared to other tissues. Publications confirmed the human and/or mouse heart-specific expression of FHL2 [59], [60], HSPB7 [61], [62], and MYOZ2 [63], and PCR confirmed the heart specific expression of TNNT2, FHOD3, PLN, MYH6, CSRP3, NPPA, RYR2, and PRUNE2.

FHL2 functions in many fundamental processes by interacting with a variety of types of proteins including structural proteins, kinases, and transcription factors. HSPB7 interacts with alpha filamin, and is potentially involved in chaperone activity and maintenance of the cytoskeletal network in the cardiac muscle. MYOZ may serve as intracellular binding proteins involved in linking Z line proteins and localizing calcineurin signaling to the sarcomere. FHOD3 is an actin-organizing protein that may regulate stress fiber formation. PLN has been postulated to regulate the activity of the calcium pump of the cardiac sarcoplasmic reticulum. CSRP3 is an organizer of cytosolic structures in cardiomyocytes. Mutations in this gene may cause hypertrophic cardiomyopathy and dilated cardiomyopathy in humans [64]. NPPA is a hormone playing a key role in cardiovascular homeostasis. Both MYH6 and RYR2 are responsible for cardiac muscle contraction. RYR2 mutations may cause catecholaminergic polymorphic ventricular tachycardia (CPVT) and arrhythmogenic right ventricular dysplasia (ARVD) [65], [66].

Full length PRUNE2 contains 3,088 amino acids, which can be divided into 19 exons. PRUNE2 has 5 isoforms: BMCC1 [Bcl-2/adenovirusE1B 19 kDa-interacting protein (BNIP) 2 and Cdc42 GAP homology (BCH) motif-containing molecule at the C-terminal region1], BNIPXL (BNIP2 extralong), C9orf65 (Chromosome 9 Open Reading Frame 65), PRUNE2, and Olfaxin. BMCC1 is associated with neuronal apoptosis [67], BNIPXL is an N-terminal truncated form of BMCC1 and is related to cellular transformation [68], C9orf65 is a biomarker that distinguishes leiomyosarcomas from gastrointestinal stromal tumors [69], PRUNE2 is a binding protein of 8-oxo-GTP that contains C9orf65 and BMCC1 [70], [71]. These four PRUNE2 isoforms may play crucial roles in Alzheimer’s disease and cancer. Olfaxin is the most recently discovered PRUNE2 isoform that is located in the olfactory systems [72].

Neuronal tissues were not included in our samples. The expression of PRUNE2 was significantly greater in the heart compared to other tissues in the mouse (*P*<0.001) but not human (*P*<0.5) Searching NCBI PubMed returned no publications about PRUNE2 in the heart. However, our semi-quantitative and real-time quantitative PCR confirmed the high expression of PRUNE2 in both human and mouse heart.

From GEO profiles we found that expression of PRUNE2 is decreased in ERRα-deficient mouse hearts. Considering ERRα is an orphan nuclear receptor that plays a critical role in regulating cellular energy metabolism, the decreased expression of PRUNE2 in ERRα-deficient mouse hearts suggests that PRUNE2 may be involved in energy metabolism in the mouse heart.

PRUNE2 is likely to be related to heart diseases. The expression of PRUNE2 is decreased in Lmna H222P mutants, which in turn are related to Emery–Dreifuss muscular dystrophy [13]. These results suggest an association between PRUNE2 and Emery–Dreifuss muscular dystrophy.

The function of PRUNE2 in cardiomyocyte diseases is linked to serum response factor (SRF). SRF plays a critical role in mesodermal development [73] and the deletion of SRF causes embryonic lethal cardiovascular phenotypes. Expression of PRUNE2 is upregulated in SRF-null mutants, which indicates that PRUNE2 may be associated with cardiomyocyte diseases. Increased expression of PRUNE2 is also found in patients with idopathic dilated heart failure and ischemic heart failure, which supports the role of PRUNE2 in heart function. PRUNE2 expression is the highest in the hearts of genetically hypertensive BPH inbred strains of mice compared to the normotensive BPN and hypotensive BPL inbred strains. All of these data suggest an important role for PRUNE2 in heart function and heart diseases.

### Muscle-Specific Genes

All 8 of the muscle specific genes we identified are well-studied for their roles in muscle function: MYOT (myotilin), TNNC2 (troponin C type 2, fast), TNNI2 (troponin I type 2, skeletal, fast), TNNT3 (troponin T type 3, skeletal, fast), MYBPC1 (myosin binding protein C, slow type), MYOZ1 (myozenin 1), ACTN3 (actinin, alpha 3), and MYBPC2 (myosin binding protein C, fast type). We confirmed the muscle-specific expression of TNNC2, TNNI2, TNNT3, ACTN3, MYBPC1, MYBPC2, and MYOZ1 by PCR and two publications supported the muscle-specific expression of MYOT [74], [75].

MYOT is located within the Z-disc of sarcomeres. Mutations in the MYOT gene cause various forms of muscular dystrophy [76], [77], [78], [79]. Troponin is a key protein controlling striated muscle contraction. It is composed of 3 subunits: the TNNI subunit inhibits actomyosin ATPase, the TNNC subunit binds to calcium and overcomes the inhibitory action of the troponin complex on actin filaments, and the TNNT subunit binds to tropomyosin and TNNC. TNNI2, TNNC2, and TNNT3 are specifically expressed in muscle, whereas TNNT2 is mainly expressed in cardiac muscle. MYBPC1 and MYBPC2 are skeletal muscle slow-twitch and fast-twitch myosin binding proteins, which can regulate the activity of actin-activated myosin ATPase to modulate muscle contraction. MYOZ1 functions in modulating calcineurin signaling in skeletal muscle.

### Adipose-Specific Genes

Six genes that were confirmed by publications to be highly expressed in human and mouse adipose tissue are: RETN (resistin) [80], [81], [82], ADIPOQ (adiponectin, C1Q and collagen domain containing) [83], LEP (leptin) [84], [85], PPARγ (peroxisome proliferator-activated receptor gamma) [86], [87], CIDEC (Cell death–inducing DFF45-like effector C) [88], CCDC80 (coiled-coil domain containing 80) [89], and DGAT2 (diacylglycerol O-acyltransferase 2) [90]. Our PCR data showed the adipose specific expression of CIDEC (cell death-inducing DFFA-like effector c) and ACVR1C.

Both RETN and ADIPOQ are adipokines that control insulin sensitivity and fat metabolism. LEP controls the size of adipose depots by affecting food intake and energy expenditure. PPARγ is a well-known regulator of adipocyte differentiation, glucose hemeostasis, and blood pressure. CIDEC is localized around the lipid droplet in adipocytes and regulates lipid droplet formation [91], [92]. It can regulate energy balance and obesity [93] and induce cell apoptosis [94]. CCDC80 is a secreted protein that regulates adipocyte differentiation [95], whereas DGAT2 is an enzyme that catalyzes the final step of mammalian triglyceride synthesis [96] and may be involved in the mechanisms of obesity, insulin resistance, and leptin resistance.

Our SV and fat cell fractionation studies showed that ACVR1C is adipocyte-specific, but not preadipocyte-specific. ACVR1C was further confirmed to be both WAT-specific and BAT-specific. When fasted, the expression of ACVR1C increases significantly in rat WAT and BAT, which suggests a role for ACVR1C in fat metabolism. In the adipose tissue from obese diabetic women, the expression of ACVR1C is decreased significantly, which suggests a role for ACVR1C in obese diabetic symptoms.

In summary, our approach provides a new and powerful procedure to discover novel tissue-specific genes and predict the processes or pathways in which they may be involved. With this method, we discovered novel tissue-specific genes: AMDHD1 in the liver, PRUNE2 in the heart, and ACVR1C in the adipose tissue. Our procedure also can be extended to other tissues in other species. This approach is an efficient way of integrating valuable databases to identify novel sets of tissue-specific genes that are related to tissue growth and development, and diseases.

## Supporting Information

Table S1 **Tissue-specific gene expression values based on GEO DataSet(GDS)596 for the human and GDS3142 for the mouse.** The microarray gene expression profiles for the six tissues (kidney, liver, lung, heart, muscle, and adipose tissue) of the human and mouse were collected from GEO DataSet(GDS) in the National Center for Biotechnology Information (NCBI) web page. *Average gene expression values for each tissue including a target tissue were obtained. **Those average values for each tissue were divided by an average value of a target tissue and then averaged for obtaining one representative value. If the representative value is lower, then tissue-specificity for a target tissue is higher because it was divided by an average value of a target tissue. ***An alternative approach shows that an average value of a target tissue was divided by average of averages for each tissue. If the result value is higher, then tissue-specificity for a target tissue is higher because an average value of a target tissue was divided. Figure 1 shows the schematic diagram for the above procedures.
(XLSX)
